# Bridging epidemiology with population genetics in a low incidence MSM-driven HIV-1 subtype B epidemic in Central Europe

**DOI:** 10.1186/s12879-015-0802-6

**Published:** 2015-02-15

**Authors:** Maja M Lunar, Anne-Mieke Vandamme, Janez Tomažič, Primož Karner, Tomaž D Vovko, Blaž Pečavar, Gabriele Volčanšek, Mario Poljak, Ana B Abecasis

**Affiliations:** Institute of Microbiology and Immunology, Faculty of Medicine, University of Ljubljana, Ljubljana, Slovenia; KU Leuven - University of Leuven, Department of Microbiology and Immunology, Rega Institute for Medical Research, Clinical and Epidemiological Virology, B-3000 Leuven, Belgium; Unidade de Microbiologia e Global Health and Tropical Medicine, Instituto de Higiene e Medicina Tropical, Universidade Nova de Lisboa, Lisbon, Portugal; Department of Infectious Diseases, University Medical Center Ljubljana, Ljubljana, Slovenia; Unidade de Saúde Pública Internacional e Bioestatística e Global Health and Tropical Medicine, Instituto de Higiene e Medicina Tropical, Universidade Nova de Lisboa, Lisbon, Portugal

**Keywords:** HIV-1, Subtype B, Molecular epidemiology, Phylogeny, Phylodinamics, Substitution rate

## Abstract

**Background:**

The HIV-1 epidemic in Slovenia, a small Central European country, has some characteristics that make it an ideal model to study HIV-1 transmission. The epidemic is predominantly affecting men who have sex with men infected with subtype B (89% of all patients), has a low prevalence (less than 1/1000) and is growing slowly. The aim of the present study was to analyze in detail the evolutionary history and the determinants of transmission.

**Methods:**

A total of 223 partial *pol* gene sequences from therapy naïve individuals were included, representing 52% of all patients newly diagnosed in 13 years (2000–2012) and analyzed together with genetically similar worldwide sequences, selected in a BLAST search.

**Results:**

Combined analysis (maximum likelihood and Bayesian) of HIV-1 transmission chains revealed 8 major clusters (n ≥ 10 patients), 1 group of 4 patients, 2 trios and 12 transmission pairs, thus leaving only 43 (19.3%) Slovenian patients infected with subtype B without a local epidemiological link, indicating a predominance of local transmission of HIV-1 infection. Bayesian analysis performed on a full set of sequences estimated several introductions of HIV-1 into Slovenia, with the most recent common ancestor (tMRCA) of the earliest Slovenian cluster dated to the late 1980s, although tMRCAs obtained from separate independent analysis of each cluster showed considerably more recent estimates. These findings indicate inconsistencies in molecular clock estimation, which we further explored.

We hypothesize that these inconsistent dating estimates across the tree could be caused by an evolutionary rate acceleration of HIV-1 after entering the Slovenia epidemic that is not taken into account by the molecular clock model. It could be caused by a lower transmission rate in this setting, as demonstrated by the low epidemic growth rate estimated by Bayesian skyline plot analysis.

**Conclusions:**

HIV-1 subtype B was introduced into Slovenia at several time points from the late 80s onward. The majority of patients had a local transmission link, indicating a closed HIV community. The observed slower epidemic rate suggests that individuals with a long-lasting infection are the driving force of the epidemic in this region.

## Background

Viral sequence data, particularly in the case of HIV-1, which has extremely high mutation rates, can help characterize the evolutionary history and population genetics of an epidemic (reviewed in Pybus and Rambaut 2009). By linking HIV evolution to the dynamics and transmission of the infection, phylogenetic tree reconstruction and the coalescent can help to indicate the nature of transmission events that happened in the past. By combining it with molecular clock models, it can help to time the origin of epidemics and reconstruct changes in pathogen population size over time [1].

Extensive studies have been undertaken on HIV-1 subtype B sequences with the aim of understanding local epidemic patterns. Lewis et al. (2008) analyzed subtype B sequences obtained from patients (predominantly men who have sex with men (MSM)) attending a clinic in London between 1997 and 2003. The dated phylogenies indicated that the majority of transmissions within clusters occurred between 1995 and 2000. Furthermore, 25% of transmissions happened within 6 months after infection, thus showing an episodic pattern of the HIV epidemic among MSM in London [2]. A similar study was conducted in 2011 on a vast set of 14,560 sequences from London, in which 52% of individuals were determined to have a transmission link to at least one other individual. A transmission interval of less than 6 months was estimated among 16% of individuals, whereas the median internode interval of the entire set was 22 months. This confirmed an important role of recent infection in transmitting the infection forward in this predominantly MSM group [3]. A study examining subtype B sequences of the MSM epidemic in Italy, Slovenia’s neighbor, revealed several introductions of the virus into the country. Half of the characterized clusters originated in the early 1990s, about 20 years later than in other Western European countries. Demographic analysis showed a rapid exponential increase in the effective population, starting in the early 1980s and reaching a plateau 10 years later [4]. On the other hand, a study focusing on the HIV subtype B epidemic in the Balkan area observed multiple introductions of HIV and, through dated phylogeny, suggested that the epidemiological network did not exist before the early 1970s. One of the first clades proposed to emerge at this time was of Slovenian origin. Slovenia was also found to be the probable entry point of HIV subtype B into the Balkans, since it was frequently determined as the most recent common ancestor (MRCA) location of Balkan clades [5].

Slovenia is a small Central European country with a modest burden of HIV disease, with less than 1 per 1000 inhabitants infected. The first AIDS case in the country was registered in June 1986 and, by the end of 2012, 592 HIV cumulative cases had been reported [6,7]. The HIV epidemic mostly affects MSM and subtype B is the most prevalent subtype, with 84% of patients diagnosed in the period 1996–2005 and 89% in 2005–2010 [8,9]. The characteristics of the subtype B epidemic in Slovenia were initially investigated in a previous study conducted in 2006, examining a 10-year period from 1996 to 2005. Phylogenetic analysis determined 14 potentially significant clusters, comprising 5, 4, or 3 patients and 11 transmission pairs, mostly among MSM (79%). Factors found significantly associated with clustering were: infection during or after 2003, diagnosis of primary HIV infection, higher CD4 cell count, and the infection being acquired in Slovenia [10].

The aim of this study was to determine the key properties of the subtype B epidemic in Slovenia, the traits associated with the clustering of individuals and to characterize the start of the HIV local epidemic and its course over time. Given the high proportion of subtype B in Slovenia (88.9%), only sequences of this subtype were included in our study, which still amounted to more than half of all patients diagnosed in the whole country.

## Methods

For the purpose of this study, data and sequences were gathered from 3 previous studies conducted in Slovenia examining the prevalence of transmitted drug resistance among therapy naive HIV-1 positive patients diagnosed in the years 2000–2004, 2005–2010 and 2011–2012, approved by the Medical Ethics Committee at the Ministry of Health of Slovenia (Approval Ref. No.: 126/12/03) [9,11,12]. The following epidemiological and laboratory data were available for statistical analysis: gender, age at time of diagnosis, year of diagnosis, country of origin, acute retroviral syndrome (ARS), Centers for Disease Control and Prevention (CDC) class, AIDS defining illnesses, other sexually transmitted diseases, most probable route of HIV infection, relationship with the source of HIV infection (sex with anonymous person or stable relationship), country where the infection most probably occurred, viral load and CD4 cell count at the time of diagnosis and presence of surveillance for drug resistance mutations (SDRMs).

For the purpose of the incidence estimate, patients were estimated as recently infected when diagnosed and sampled within 155 days after infection, or as having a long-standing infection (LSI) when diagnosed after 155 days following infection. In brief, patients with a baseline CD4+ cell count of fewer than 200 cells/mm^3^ and/or with a baseline viral load (VL) of fewer than 400 copies/ml were first automatically characterized as having a LSI. For the remainder, the Aware™ BED™ EIA HIV-1 Incidence Test (BED test) (Calypte Biomedical Corporation, Portland, Oregon) was employed on a sample taken within 3 months of diagnosis, according to the manufacturer’s instructions. Briefly, the principle of the BED test is as follows: patients’ plasma samples were first diluted 1:101 and added to a goat-anti–human IgG coated microplate, capturing anti–HIV IgG and non–anti–HIV IgG from the samples. The amount of specific anti-HIV-1 antibodies was proportional to the optical density (OD) values obtained by spectrometric analysis. Normalized optical density was calculated (OD_n_ = plasma sample OD/calibrator OD) and, on the basis of the defined OD cut-off values of the assay, the patients were classified as having a LSI or RI, with the suggested window period of infection being 155 days [13]. This determination was possible for 213/223 Slovenian patients included in the study (for the remaining 10 patients a plasma sample taken within 3 months after HIV-1 diagnosis was not available).

Sequences were re-analyzed for subtype determination by employing the REGA HIV-1 Automated Subtyping Tool, version 2 [14]. Only subtype B sequences were selected for this study, a total of 223 partial *pol* gene sequences, exhibiting an inclusion of 53% ± 15% of newly diagnosed patients in the study per year.

Alignments were made using ClustalW, available in the BioEdit package, and edited and trimmed to 953 base pairs [15]. A quick neighbor joining (NJ) tree was created using Seaview with 100 bootstrap replicates [16]. A subset of Slovenian sequences was selected from all parts of the NJ tree by visual inspection, to represent the complete diversity of the epidemic. Depending on the size of the cluster, at least three to five sequences per transmission cluster were included. These sequences were then used to search the GenBank public database for other closely related sequences (controls), using the BLAST search tool [17]. The 10 most similar sequences per Slovenian sequence were retrieved from GenBank. Since population genetics analysis is only possible using sequences that contain sampling time information, only sequences with an available sampling time were selected for controls. The majority of Slovenian sequences gave similar BLAST results, thus a fair amount of control sequences were found to be repeated. After removing these duplicate sequences, only 84 control sequences remained, together with Slovenian sequences forming a dataset of 307 subtype B sequences for further analysis.

jModeltest software was employed for selection of the best fitted evolutionary model to be used on the selected dataset, with an additional three subtype A1 sequences (accession numbers AB098332, AB253421, AB285785), selected to root the obtained maximum likelihood (ML) tree [18,19]. Using jModelTest, TVM + I + G was determined as the best fitted evolutionary model for the data. Since this evolutionary model is not available in most phylogenetic tree construction software, the selection of the closest model, based on the hierarchy of evolutionary models provided by jModeltest developers, was used instead. Thus, all additional analyses (maximum likelihood and Bayesian probability) were run using the next simpler model, HKY + I + G. This model, when combined with 2 codon partitions (1 + 2 codon, 3 codon), has been previously determined as performing best for most protein viral datasets [20]. The ML phylogenetic tree was constructed using a sub-tree pruning and regrafting + nearest neighbor interchange (SPR + NNI) search criterion, as implemented in PhyML 3.0 software [21]. The obtained phylogeny was visualized using FigTree v1.3.1 [22]. Transmission clusters were at this point identified according to the approximate likelihood ratio test (aLRT) branch support values obtained (>0.90) by the ML method.

The temporal signal of the dataset was assessed using Path-O-Gen, which showed an r-squared value of 0.53 with the best-fitting root (date range 29 years) [23].

The Bayesian analysis was performed on all Slovenian subtype B sequences and control sequences (full analysis). Additionally, separate analyses were executed on all major Slovenian clusters (≥10 Slovenian sequences) for two reasons: 1) to compare the tMRCA values obtained in the independent clusters analysis with the tMRCA values obtained based on the full data set analysis and 2) to obtain the population growth rate of each cluster. The tMRCAs of the full dataset and of major clusters were determined by the Monte Carlo Markov chain (MCMC) method available in the BEAST package v1.7.1, using a relaxed clock model with uncorrelated lognormal distribution and the Bayesian skyline coalescent model [24]. The analysis was run using a HKY + I + G substitution model and 2 codon partitions (1 + 2 codon, 3 codon) were added. The output results of BEAST were examined in Tracer v1.5 [25] and the MCMC chain was run until the effective sample size (ESS) values for all parameters exceeded 200. Convergence was achieved after 500,000,000 and 100,000,000 generations, for the full analysis and for analyses based only on cluster strains, respectively. All the analyses were repeated and the obtained duplicate results then combined using LogCombiner v1.6.1, available in the BEAST package [24]. TreeAnnotator v1.7.1 (BEAST package) was employed to remove a burn-in of 10% of all sampled trees. A Maximum Clade Credibility Tree was finally visualized and annotated using FigTree v1.3.1 [22].

The final clusters were defined by reviewing clusters previously identified in the ML analysis (aLRT > 0.90) according to posterior probability values obtained in Bayesian analysis. Only clusters with posterior probability >0.990 were then selected. Thus, cluster definition relied on both aLRT (>0.90) and posterior probability (>0.990) values. Moreover, a sensitivity analysis was conducted by altering aLRT and posterior probability cut-offs. When a higher cut-off was applied, namely, aLRT >0.95 and posterior probability >0.999, all the defined clusters remained. On the other hand, when lowering the cut-offs to aLRT >0.85 and >0.80 one of the clusters had 3 additional Slovenian sequences and one additional Slovenian cluster was identified. Regarding posterior probability, no differences were observed when lowering the cut-off, since the values on the outer nodes were much lower than 0.9, giving credibility to the selected cluster definition.

The obtained tMRCA values were compared to epidemiological data (e.g., time of infection) gathered from questionnaires, confirmed epidemiological links and compared to the results of an HIV-1 incidence algorithm.

Statistical analyses were conducted using the on-line statistical package Epi Info™ Version 3.5.3 and P ≤ 0.05 values were considered to be significant [26].

## Results

### Most Slovenian subtype B infected patients belong to large local MSM transmission clusters

The majority of patients included in the study were males (94.2%), with a mean age of 37.9 years and reporting homosexual contact (83.4%) and sex with anonymous person (52.5%) as the most probable route of HIV acquisition. Mean viral load of 4.99 (±0.979) log copies/ml and CD4 cell count of 325 (±218) cells/mm^3^ were measured at the time of diagnosis and 26.9% of patients were determined as recently infected by incidence algorithm.

According to the maximum likelihood phylogenetic tree, 8 clusters with ≥10 Slovenian sequences, 3 trios and 11 pairs of patients were identified with high support (aLRT values >0.90). Bayesian analysis was repeated in duplicate on the same set of sequences. Seven out of the 8 clusters obtained by the ML method were confirmed by the Bayesian analysis, according to posterior probability values (≥0.990). The cluster not confirmed in the further Bayesian analysis was Cluster 5, which was broken down into two smaller clusters harboring 10 and 4 patients. Branch support values for the 8 major clusters obtained in ML and Bayesian analysis can be seen in Table 1, together with maximum pairwise genetic distances (a criterion not employed for cluster identification). Alternating to different cluster cut-offs (aLRT from 0.8–0.95 and posterior probability from 0.800–0.999) did not drastically change the cluster composition. In addition, the observed transmission pairs and trios of Slovenian patients were also confirmed, with the exception of one trio, which did not reach an adequate posterior probability (0.8455). Eight major clusters (n ≥ 10 patients), 1 group of 4 patients, 2 trios and 12 transmission pairs were therefore finally identified by combining the results of maximum likelihood and the Bayesian method. Among 223 included individuals, 146 (65.5%) patients thus belonged to large transmission clusters comprising 10 or more individuals and 34 (15.2%) patients to small clusters of 2–4 patients. Thus, for only 43 (19.3%) of the Slovenian patients infected with subtype B no local epidemiological link was observed by phylogenetic inference (Table 2).

**Table 1:** Genetic distance, aLRT and posterior probability of 8 major clusters of Slovenian sequences and substitution rate (*10 ^**-2**^ **) obtained by employing Bayesian analysis on two different sets of sequences; set comprised of all Slovenian subtype B sequences and separate sets of only clustered sequences**

	**Maximum pairwise genetic distance** ^**2**^	**aLRT** ^**2**^	**Posterior probability** ^**3**^	**Substitution rate - full analysis** ^**3**^			**Substitution rate - analysis based on cluster strains** ^**3**^		
				**Mean**	**Median**	**95% HPD**	**Mean**	**Median**	**95% HPD**
**Cluster 1**	2.38%	0.986	1	3.20	2.85	0.80–6.53	1.71	1.70	1.24–2.22
**Cluster 2**	2.56%	0.991	1	3.85	3.55	1.29–7.34	1.41	1.38	0.76–2.14
**Cluster 3**	1.85%	0.985	0.9998	2.19	2.02	0.76–4.00			
**Cluster 4**	2.12%	0.999	0.9999	2.74	2.49	0.92–5.23			
**Cluster 5** ^**1**^	3.14%	0.928	0.9996	2.46	2.16	0.50–5.27			
**Cluster 6**	3.73%	0.981	1	2.46	2.19	0.63–4.97			
**Cluster 7**	1.32%	0.972	1	2.06	1.87	0.60–3.86			
**Cluster 8**	1.01%	0.999	1	2.41	2.25	1.04–4.29			

NOTE: The Bayesian analysis was performed in duplicate by using a relaxed clock model with uncorrelated lognormal distribution and Bayesian skyline coalescent model.

^1^numbers are shown for the newly defined Cluster 5 with only 10 patients.

^2^values obtained by maximum likelihood analysis.

^3^values obtained by Bayesian analysis.

aLRT = approximate likelihood ratio test branch support values, HPD = highest posterior density.

**Table 2:** **Characteristics of patients included in the analysis and comparison between patients found within a large cluster (≥10 patients) or with a transmission link to patients without these observed connections for determined associations with P-value ≤ 0.1**

		**Total population**	**%**	**In cluster (n ≥ 10)**	**%**	**Not in cluster**	**%**	**P-value** ^**2**^	**With a local transmission link (n = 2–39)**	**%**	**No link**	**%**	**P-value** ^**2**^
Patients													
		223	52.2%^1^	146	65.5%	77	34.5%		180	80.7%	43	19.3%	
Gender													
	Male	210	94.2%	141	67.1%	69	32.9%	0.0761	170	81.0%	40	19.0%	0.9488
	Female	13	5.8%	5	38.5%	8	61.5%		10	76.9%	3	23.1%	
Year of diagnosis													
	2000–2004	45	20.2%	23	51.1%	22	48.9%	**0.0388**	31	68.9%	14	31.1%	**0.0479**
	2005–2008	86	38.6%	60	69.8%	26	30.2%	0.3555	74	86.0%	12	14.0%	0.1514
	2009–2012	92	41.3%	63	68.5%	29	31.5%	0.5178	75	81.5%	17	18.5%	0.9391
Country of origin													
	Slovenia	174	78.0%	122	70.1%	52	29.9%	0.2919	148	85.0%	26	15.0%	**0.0479**
	Other	15	6.7%	8	53.3%	7	46.7%		9	60.0%	6	40.0%	
	Unknown	34	15.3%	16	47.1%	18	52.9%		23	67.6%	11	32.4%	
Origin of the virus^3^													
	Slovenia	131	58.7%	105	80.2%	26	19.8%	**<0.0001** ^**4**^	120	91.6%	11	8.4%	**<0.0001** ^**4**^
	Other	36	16.1%	11	30.6%	25	69.4%		19	52.7%	17	46.3%	
	Unknown	56	25.1%	30	53.6%	26	46.4%		41	73.2%	15	26.8%	
SDRMs													
	Detected	8	3.6%	2	25.0%	6	75.0%	**0.0431**	3	37.5%	5	62.5%	**0.0153**
	Not detected	215	96.4%	144	67.0%	71	33.0%		177	82.3%	38	17.7%	

^1^proportion among all newly diagnosed HIV-1 patients in the period 2000–2012 in Slovenia.

^2^for significance testing, the Fisher exact test was employed for categorical data and t-statistics for continuous data. P-values ≤ 0.05 are shown in bold.

^3^the origin of the virus is determined according to the country where the infection most probably occurred, as reported in the patients’ questionnaires.

^4^significant characteristic after employing a Bonferroni correction, at a significance level of <0.0033.

SDRMs = surveillance for drug resistance mutations, n = number of patients in a transmission cluster.

Statistical analysis examining the characteristics of patients found in large clusters (≥10 patients) revealed that significantly fewer patients were diagnosed prior to 2005 (p = 0.0388), significantly more patients reported Slovenia as the country in which the infection occurred (p < 0.0001) and significantly fewer patients in clusters were carrying drug resistance mutations (DRMs) (p = 0.0431). In addition, statistical analysis was repeated by joining patients belonging to small clusters (2–4 individuals) with the 146 included in large transmission clusters, totaling 180 (80.7%) patients with an observed transmission link to another Slovenian patient. The results again showed a Slovenian origin of the virus as a trait significantly associated with patients having a determined transmission link (p < 0.0001), fewer patients diagnosed prior to 2005 had a transmission link detected (p = 0.0479) and fewer patients with DRMs were found among patients with a transmission link (p = 0.0153). Additionally, more patients were found to have a transmission link among those reporting Slovenia as their country of origin than patients of foreign nationality (p = 0.0479), an effect not seen when observing only large clusters. Results of statistical analysis with the obtained P-values of <0.1 can be seen in Table 2. In addition, the following characteristics were also compared between the groups but showed no significance: age at time of diagnosis, acute retroviral syndrome (ARS), CDC class, AIDS defining illnesses, other sexually transmitted diseases, most probable route of HIV infection (homo/bisexual, heterosexual or other), relationship with the source of HIV infection (sex with anonymous person or stable relationship), viral load and CD4 cell count at the time of diagnosis and duration of HIV infection at the time of diagnosis as determined by incidence algorithm (recent or long-standing).

The only characteristic significantly associated with patients having a determined local transmission link after employing a Bonferroni correction (at a significance level of <0.0033) was found to be Slovenia as the country in which the infection occurred.

**Figure 1:**
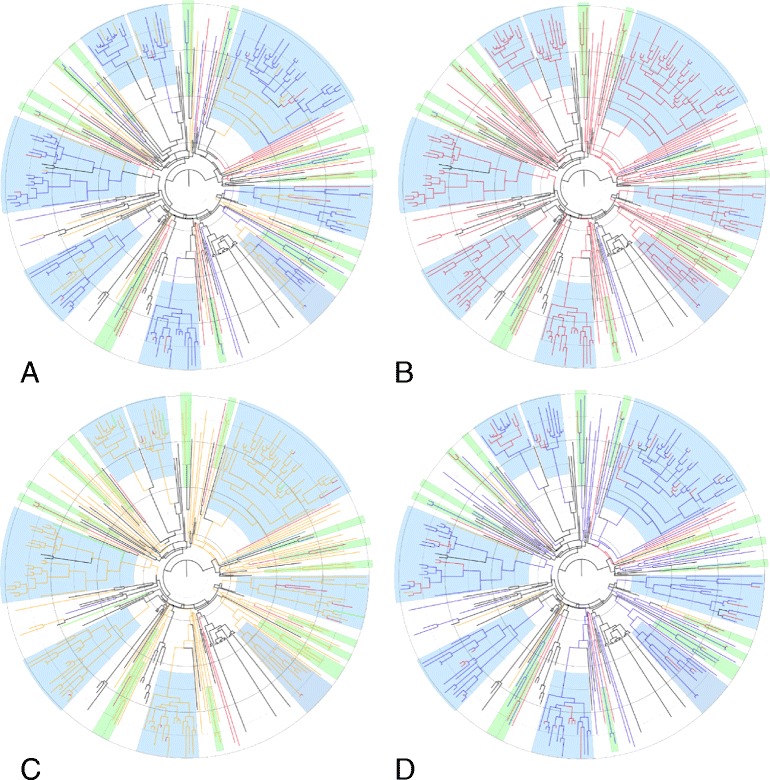
**Bayesian maximum clade-credibility trees of the Slovenian subtype B epidemic.** Defined major clusters (n ≥ 10 patients) are highlighted in blue and small clusters (n = 2–4 patients) in green. The branches of the Slovenian sequences are colored according to the patients’ characteristics: **(A)** Country of infection: blue = Slovenia, red = abroad, yellow = unknown; **(B)** Gender: red = male, blue = female; **(C)** Mode of HIV-1 acquisition: yellow = homosexual contact, red = heterosexual contact, blue = injection drug use, teal = vertical transmission, green = unknown; **(D)** Timing of infection: blue = long-standing infection, red = recent infection, yellow = unknown. The branches of the control sequences are depicted in black. The rings are set at 5-year intervals with the outer circle starting at the sampling time of the latest sequence (2012.97).

The network of patients analyzed can also be seen in the obtained combined and annotated (10% burn-in) Bayesian phylogenetic trees (Figure 1). It can be seen that 6 out of 8 large clusters contained at least one patient reporting that he or she was infected abroad, which was also true for almost half of small clusters. All identified clusters harbored at least 2 patients previously identified as having been recently infected at diagnosis (according to the BED test). Clusters were primarily composed of patients reporting homosexual contact as the mode of HIV acquisition. Five clusters contained only male patients, even though they included some reports of heterosexual transmission routes. This indicate either a missing female transmission link or false statements about mode of transmission. Most of the heterosexual transmissions seem to be limited to small clusters, with only a few in large clusters. Of the 2 patients reporting injection drug use, 1 male infected his female partner (derived from epidemiological data), but an onward spread of the virus was not noted. By visual inspection of the phylogenetic trees it was observed that GenBank control sequences were mostly paraphyletic to Slovenian clusters, indicating a potential geographical origin for those clusters (Figure 1). Sequences found most closely related to large clusters of Slovenian sequences were derived from USA, Germany, Belgium, Canada and France (data not shown).

### The Slovenian HIV-1 epidemic started in the 1980s

For an estimation of the time to the most recent common ancestor (tMRCA) of Slovenian sequences, Bayesian analysis was performed on different datasets, always in duplicate. The 2 runs were combined and the obtained Bayesian phylogenetic trees are seen in Figure 1 and the corresponding tMRCA values of 8 major clusters in Table 3. According to mean values, the earliest dated cluster of Slovenian sequences is Cluster 6, dating the start of local transmission of the Slovenian HIV-1 epidemic to 1986 (95% highest posterior density (95% HPD): 1981.4–1990.5) (Table 3).

**Table 3:** **Times to the most recent common ancestor (tMRCAs) of 8 major clusters of Slovenian sequences obtained by employing Bayesian analysis on two different sets of sequences; set comprised of all Slovenian subtype B sequences and separate sets of only clustered sequences**

	**Full analysis**			**Analysis based on cluster strains**				
	**Mean**	**Median**	**95% HPD**	**Mean**	**Median**	**95% HPD**	**Sampling dates**	**Time depths** ^**2**^
**Cluster 1**	1990.6	1991.4	1987.1–1996.1	1998.0	1998.3	1995.2–2000.4	2001.42–2012.73	22.13
**Cluster 2**	1986.9	1988.1	1983.1–1992.9	1990.0	1990.8	1980.1–1998.1	2003.06–2012.68	25.78
**Cluster 3**	1999.1	1999.9	1995.4–2002.9				2005.46–2012.74	13.64
**Cluster 4**	1991.4	1992.2	1987.0–1998.1				2002.40–2012.64	21.24
**Cluster 5** ^**1**^	1990.6	1991.1	1987.1 – 1995.6				2000.68–2009.47	18.87
**Cluster 6**	1986.1	1987.4	1981.4–1990.5				2000.25–2012.92	26.82
**Cluster 7**	2003.0	2003.6	2000.8–2005.3				2005.32–2012.45	9.45
**Cluster 8**	2001.2	2001.7	1999.0–2003.4				2004.01–2011.77	10.57

**Note:** The analysis was performed in duplicate by using a relaxed clock model with uncorrelated lognormal distribution and Bayesian skyline coalescent model.

^1^numbers are shown for the newly defined Cluster 5 with only 10 patients.

^2^time depths are shown in years and calculated according to the mean years of MRCA obtained in the full analysis.

HPD = highest posterior density.

Separate MCMC runs were executed for each of the eight identified Slovenian transmission clusters (including the newly defined Cluster 5 with 10 individuals) for two reasons: 1) to compare the tMRCA values obtained in the independent clusters analysis with the tMRCA values obtained based on the full analysis previously described and 2) to obtain the population growth rate of each cluster. As seen in Table 3, the analysis reached convergence only for Clusters 1 and 2.

Additionally, substitution rates obtained in the full analysis were compared to rates obtained in independent analyses based on cluster strains (Table 1).

### tMRCAs estimated by Bayesian analysis generally precede the estimated time of infection determined by the BED assay

**Figure 2:**
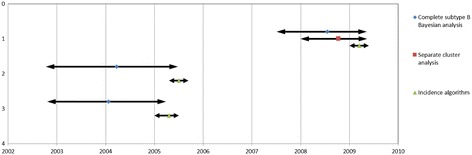
**Times of infection of epidemiologically linked patients and the corresponding tMRCAs (95% HDP intervals).** Times of infection were estimated according to the results of the incidence algorithm incorporating the Aware™ BED™ EIA HIV-1 Incidence Test (BED test) (Calypte Biomedical Corporation, Portland, Oregon). Patients were estimated as recently infected when diagnosed and sampled within 155 days after infection, or as having a long-standing infection (LSI) when diagnosed after 155 days following infection. When patients were determined as recently infected, the suggested time of infection therefore occurred in a window period of 155 days. For transmission pair 1, only one patient was recently infected. The tMRCAs were obtained following BEAST analysis.

Additional information of a reported transmission link was available for some patients included in this study. Together with information of recent infection provided by the incidence algorithm, it allows a fairly reliable estimate of the time of infection. The mentioned data were available for 2 pairs of patients, in which at least one of the two patients was determined as recently infected and one trio of patients, all infected recently (Table 4). The BED assay, used to determine whether an infection was acquired recently, employs a 155-day time interval following infection for characterization of a recent infection. For this reason the time of infection for these individuals defined as being recently infected was thus estimated to be within a 155-day interval prior to the time of sampling. Since all the patients were part of previously identified clusters, tMRCA results were compared between the Bayesian analysis of the complete set of 307 sequences and separate analyses based on cluster sequences. In Table 4 and Figure 2 (mean values are depicted with markers and 95% HPD intervals with arrows), it can be seen that the 95% HPD values estimated by Bayesian analysis generally preceded the BED estimated time of infection. The mean tMRCA date estimated by Bayesian analysis of a complete dataset was 0.7 years, 1.3 years and 1.2 years before the estimated date of infection by BED assay for transmission pair 1, pair 2 and the trio, respectively. The median tMRCA was 0.5 years, 1.1 year and 1.1 year before the estimated date of infection by BED assay for transmission pair 1, pair 2 and the trio, respectively.

**Table 4:** **Comparison of the obtained tMRCA values from Bayesian analysis to estimated times of infection according to incidence algorithms for patients with a known source of infection**

	**Cluster affiliation**	**Incidence algorithm**	**Full analysis**			**Analysis based on cluster strains**		
			**Mean**	**Median**	**95% HPD**	**Mean**	**Median**	**95% HPD**
**Pair 1**	Cluster 1	2009.0–2009.4	2008.5	2008.7	2007.5–2009.4	2008.8	2008.9	2008.0–2009.4
**Pair 2**	Cluster 5	2005.3–2005.7	2004.2	2004.4	2002.8–2005.5			
**Trio**	Cluster 3	2005.0–2005.5	2004.1	2004.3	2002.8–2005.2			

HPD = highest posterior density.

### Separate analysis based on sequences of Slovenian clusters indicates low population growth rates

**Figure 3:**
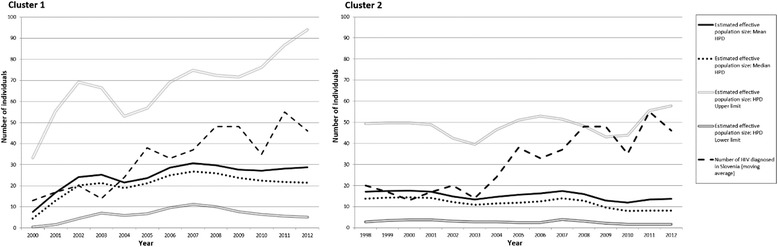
**Bayesian skyline reconstruction analysis performed for Cluster 1 and Cluster 2 to observe the population growth.** Mean, median, upper and lower 95% highest posterior densities (HPD values) of the effective population growth are depicted. A moving average of the incidence of HIV diagnosis in Slovenia per year is also added.

Since different introductions of HIV occurred in Slovenia, the effective population size should be estimated from analysis performed on cluster sequences. In order to observe the population growth, Bayesian skyline reconstruction analyses were performed for the biggest of the clusters, Cluster 1 and Cluster 2, using Tracer v1.5. The obtained graphs, showing mean, median, upper and lower 95% HPD values of effective population growth, are shown in Figure 3. The numbers of newly diagnosed HIV patients in Slovenia over the years are also included. It suggests that there has been a constant effective population size of HIV infected individuals present in Slovenia, without any drastic fluctuations. In the analysis of Cluster 1, a rise is seen from 2000 until 2003 and thereafter a slight fall. After 2004, a rise in the numbers is again seen, until 2007, when the effective population seems to have reached a peak. Comparing coalescent results to actual numbers of new HIV diagnoses, Cluster 1 shows comparable fluctuations in its values over time. The population growth seen in the analysis of Cluster 2 shows a steadier trend, with no changes seen after 1998.

## Discussion

To the best of our knowledge, this is the first nation-wide phylodynamics study examining the characteristics of an HIV-1 subtype B driven epidemic on a population consisting of over half of all HIV-positive patients diagnosed in the 13-year study period. The HIV-1 epidemic in Slovenia is mainly driven by homosexual intercourse and through local transmission, where eighty percent of patients have an identified local transmission link (meaning either being infected by someone or infecting someone locally). Several introductions of HIV-1 to Slovenia have been observed after the year 1986. Interestingly, faster substitution rate of Slovenian sequences was seen, which suggests low transmission rates and a slowly growing epidemic.

### The HIV-1 epidemic in Slovenia results from several introductions after 1986 which then propagated mainly through local transmissions

In order to understand the dynamics of HIV-1 subtype B transmission in Slovenia, we analyzed a dataset including fifty-two percent (223/427) of all newly diagnosed patients over the 13-year time span between 2000 and 2012. Since MSM are the most vulnerable population for HIV infection in Slovenia, male gender was over-represented (94% of all included patients). Indeed, a significant association between male gender and subtype B had already been determined in a previous study conducted in Slovenia [9].

The majority of infected patients belong to large transmission clusters: 65.5% of patients belonged to a transmission cluster together with 9 or more individuals. When including also smaller transmission clusters of 2–4 individuals, 80.7% Slovenian patients infected with a subtype B virus have a local transmission link. This is also the reason why few non-Slovenian sequences could be found when using the BLAST search tool to select for additional control sequences to be included in the analysis. This supports the findings that in Europe, infections are acquired predominantly from patients of the same country [27].

All the identified clusters were nested within subtype B control sequences selected from GenBank, indicating that the HIV epidemic in Slovenia is comprised of several introductions of HIV into the country. tMRCA values obtained from the analysis of the complete dataset indicate that HIV-1 subtype B was introduced into Slovenia at different time points from the late 80s onward. The earliest dated two clusters of Slovenian sequences show 1986 as the year in which HIV was introduced into the country. This was also the year when the first AIDS patient was diagnosed and reported in Slovenia, indicating consistency between epidemiological data and Bayesian tMRCA estimates. Even then, most infections were found among MSM, as they are today; this vulnerable risk group thus probably imported HIV into Slovenia at different time points.

### The HIV-1 epidemic in Slovenia is mainly driven by homosexual intercourse and through local transmission

In the current study, three factors were found to be significantly associated with patients belonging to a large cluster: the fact that the patient reported Slovenia as the country in which HIV infection occurred, diagnosis of HIV after 2004 and no DRM detected. The first was expected, is consistent with the findings of large local transmission clusters as mentioned above and is in line with the study of Frentz et al. (2013) [27]. A possible explanation for the second is that the period around 2004 was when a substantial pool of HIV infected patients had formed in Slovenia, thus making HIV acquisition within the country, as opposed to abroad, more probable. On the other hand, more recent infections within clusters can be explained by the process of cluster identification. The definition of a transmission cluster is still a topic of discussion and no consensus has been reached. In any case, since divergence is quickly accumulating within a host after infection, and given the current proposed definitions of a transmission cluster, transmission clusters with a higher number of recent infections will be preferentially selected in detriment of the ones where infections were acquired over a longer time period. In such recent infections, less divergence has accumulated and therefore evolutionary distances and eventually statistical supports of the cluster (aLRT, bootstrap, posterior probability) will be higher. In this study, since evolutionary distances were not considered as a criterion for the definition of transmission clusters, we checked the age of the cluster by investigating its depth in years. Our mean (and median) depth in years were 18.6 (20.1), which is quite comparable with the mean (or median) depth in years found in other studies, ranging between 7 and 35 years [2,4,28-36].

The third finding showing statistical significance was that most patients with DRMs were not included in transmission clusters, nor did they have a transmission link. This could imply that most transmitted drug resistance (TDR) is imported, confirming the particular importance of HIV-1 drug resistance testing of newly-diagnosed treatment-naive patients infected abroad, as is the current national strategy. Alternatively, it could indicate a lower fitness of viruses harboring TDR, with lower transmissibility of such strains.

Interestingly, 40% of patients reporting that infection happened abroad did not have a transmission link, compared to only 15% among those reporting Slovenia as their country of infection. This may be due to sampling bias, since sampling in other countries, where the infection was acquired, might not be as substantial. However, this leaves 60% with a local transmission link found, even though the infection was supposedly acquired abroad. This could mean that a large proportion of patients infected abroad further transmit the disease locally. On the other hand, it is possible that these patients were not certain of their source of infection and had actually been infected in Slovenia. Uncertainty in patients’ reports of the most probable source of transmission, especially in populations that are sexually more active, has been observed in a study by Resik et al. (2007) examining two transmission networks in Cuba [37].

More than half of the large clusters were comprised of male individuals only and, furthermore, 2 clusters had individuals reporting only homosexual contact as their route of infection. However, the other 3 male-only clusters had reported heterosexual contact and, since no female individuals were found in these clusters, this could be explained by missing transmission links due to sampling or by individuals not disclosing their true sexual orientation due to stigma. The latter was described recently in a study by Hue et al. (2014), where a 1–11% misclassification of homosexuals as reported heterosexuals was noted [38]. Most of the heterosexual transmissions seem to be limited to small clusters, without further spread of the infection, with only a few in large clusters (possibly women infected by bisexual partners). All things considered, our results show that local HIV transmission in Slovenia is mainly driven by homosexual transmission clusters.

### Discrepancies in the results obtained analyzing the complete dataset and smaller separate cluster datasets

With the intention of testing tMRCA estimates based on Bayesian methods tMRCA for a particular clade was obtained by using two different calibration strategies. Firstly, the complete set of Slovenian sequences with corresponding control sequences drawn from GenBank were used for calibrating the dates; and, secondly, only cluster strains were used for calibration. For two of the clusters, the analysis using the entire dataset showed consistently earlier tMRCA estimates compared to the analysis using cluster strains only. For all other clusters, the analysis based on cluster sequences only did not reach convergence. The discrepancies in tMRCA found for the three clusters with both estimates may be caused by dramatic fluctuations of evolutionary rate, for example because of bottleneck events or the introduction of HIV in populations with different epidemic characteristics. It has been noted before that even the relaxed clock model with uncorrelated lognormal distribution, as was used here, cannot cope with extreme differences in rates across branches. Wertheim et al. conducted an analysis on more divergent data (HIV group M), examining tMRCAs of different HIV subtypes and concluded that heterotachy was responsible for this discrepancy. Relaxed clock analysis was able to detect rate changes in a separate clade, although the molecular clock model considerably underestimated the impact of this change. Using different approaches in order to clarify these obtained discrepancies, the authors concluded that none of the approaches was able to resolve these differences [39]. Our results further corroborate such findings. It was interesting to observe wider 95% HPD intervals of the obtained clusters’ tMRCAs and substitution rates in the full analysis than in the analyses with only the cluster strains, since generally one would expect that more data generates more confident results.

For 95.5% of patients included in the study, results of an incidence algorithm characterizing patients as having a recent infection (RI) or a long-standing infection (LSI) were available. A window period of 155 days was set as a cut-off for differentiating RI from LSI, meaning that when a patient is characterized as having a RI, infection was acquired in an interval of 155 days before sampling. When comparing these “BED” estimated times of infection with the tMRCAs obtained from Bayesian analysis, we found that the obtained tMRCAs were estimated earlier in time in the full analysis, as well as in the separate analysis based on cluster sequences. One explanation for these earlier estimates relies on the definition of MRCA: the Most Recent Common Ancestor corresponds to the strain that gave origin to the infections of that cluster. Therefore, this strain should have originated before the estimated time of infection, inside the body of the patient who transmitted that strain to one of the patients in the cluster. As such, it is normal and to be expected that the estimated tMRCA will be earlier than the time of infection estimated by the BED algorithm. It therefore shows that tMRCA estimations by Bayesian methods for these clusters were credible. However, these results should be interpreted with caution, since the BED test used in the incidence algorithm does not allow individual determination of the timing of infection, due to variations in immune system response, so the stated intervals of the supposed timing of infection based on this data have limitations.

All in all, these results open discussion about the accuracy and interpretation of tMRCA estimates when analyzing large datasets that represent different epidemic settings, including bottleneck events and different transmission dynamics.

### The faster substitution rate of Slovenian sequences suggests low transmission rates, a slowly growing epidemic consistent with the small HIV-1 effective population size in Slovenian clusters

In the study of Abecasis et al. (2009), the substitution rate of the *pol* region of subtype B was estimated at 0.001 substitutions/site/year. When comparing this to the rate obtained for major clusters of Slovenian sequences, a more than 10-fold faster substitution rate of Slovenian sequences is seen [40]. This, together with the findings of a low epidemic growth rate, is in line with the observation that the evolutionary rate of HIV-1 slows down when the epidemic rate increases, e.g., in a slow epidemic with small numbers of transmissions, the evolutionary rate of the virus will be faster. The HIV epidemic is indeed still small in Slovenia, thus explaining and corroborating the finding of such a fast substitution rate in the Slovenian population of HIV-1 infected patients. It has been observed that if transmissions have occurred mostly from individuals in the early stage of infection, there is no major impact of the host immune system, so less selective pressure is applied and fewer mutations accumulate in the virus transmitted [41]. Taking this into account, this suggests that the Slovenian epidemic is probably therefore driven mostly by individuals with an established long-lasting infection.

In order to determine trends in the HIV epidemic in Slovenia, analyses reconstructing population growth were additionally carried out on the two major clusters of Slovenian HIV sequences. When observing the effective population size from the Cluster 1 analysis, a rise in the numbers was seen in 2003. Interestingly, in a previous analysis of the epidemic in 2006, sequences found in clusters were predominantly obtained from individuals infected during or after 2003. It indicates that this period was important for the spread of HIV within the country [10]. As already mentioned, this was confirmed in the present study, since patients diagnosed with HIV after 2004 were found significantly more often in a large cluster. The HIV incidence in Slovenia in 2005, in fact, was more than 3-fold higher than in 2003 (35 vs. 11 newly diagnosed patients per 1,000,000 inhabitants; P < 0.05) [42].

## Conclusions

In conclusion, this is the first nation-wide phylodynamics study of a mainly HIV-1 subtype B driven epidemic including over half of all HIV-positive patients diagnosed in the 13-year study period. Our study is also unique since we compare the phylodynamic characteristics with incidence data based on HIV-1 diagnosis over time in the same population. We found various introductions of subtype B into the country from the late 80s onward. The majority of patients had a local transmission link, indicating a closed community. With respect to the faster evolutionary rate of Slovenian sequences, and its associated slower epidemic rate, we propose this is because individuals with a long-lasting infection but unaware of their status may be the driving force of the epidemic.

### GenBank accession numbers

AJ971091, AJ971092, AJ971097-AJ971099, AJ971101, AJ971103-AJ971133, AJ971135-AJ971137, AJ971140-AJ971144, GQ398934, GQ399003, GQ399157, GQ399167, GQ399210, GQ399318, GQ399406, GQ399433, GQ399494, GQ399553, GQ399574, GQ399677, GQ399709, GQ399721, GQ399731, GQ399787, GQ399882, GQ399950, GQ399979, GQ400015, GQ400033, GQ400039, GQ400057, GQ400283, GQ400355, GQ400410, GQ400411, GQ400442, GQ400452, GQ400472, JX028303-JX028406, KF753700, KF753702, KF753703, KF753706-KF753730, KF753732-KF753736, KF753738-KF753740, KF753742-KF753746, KF753748-KF753750.
